# Exploring association of mobile phone access with positive health outcomes and behaviors amongst post-partum mothers in rural Malawi

**DOI:** 10.1186/s12884-022-04782-0

**Published:** 2022-06-13

**Authors:** Martina Anto-Ocrah, Ryan J. Latulipe, Tiffany E. Mark, David Adler, Tasneem Zaihra, Joseph W. Lanning

**Affiliations:** 1 Division of General Internal Medicine, University of Pittsburgh School of Medicine, 230 McKee Place, Suite 600, PA 15213 Pittsburgh, USA; 2 Department of Emergency Medicine, New York Presbyterian/Columbia & Cornell, 525 E 68th St Box #301, New York, NY 10065 USA; 3 Department of Pediatrics, Johns Hopkins University School of Medicine, 1800 Orleans St, Baltimore, MD 21218 USA; 4 Department of Emergency Medicine, University of Rochester School of Medicine and Dentistry, 601 Elmwood Ave, NY 14642 Rochester, USA; 5 Lahey Hospital & Medical Center, 31 Mall Rd, MA 01805 Burlington, USA; 6 The School for International Training Graduate Institute, VT Brattleboro, USA

**Keywords:** Malawi, Cell/mobile phone technology, Social determinants of health, Breastfeeding, Africa, Covid, Mhealth, Digital health, Information and communication technology (ICT)

## Abstract

**Background:**

*Access to mass media and emerging technologies* (e.g., cell phones, the internet, and social media) is a social determinant of health that has been shown to profoundly influence women’s health outcomes. In the African region, where women in rural settings with limited access to care are most vulnerable to maternal mortality and other pregnancy-related morbidities, mobile phone access can be an important and life-saving health determinant.

**Objective:**

The goal of this study was to examine the association between mobile/cellular phone ownership and health behaviors of post-partum mothers in rural Malawi.

**Methods:**

In this cross-sectional study, we recruited and consented a convenient sample of 174 post-partum mothers of 4- and 5-month-olds who were attending well-child clinics in Gowa, situated in the rural Ntcheu district of Malawi. Using logistic regression models, we hypothesized that compared to non-cell phone owners, mobile phone ownership will be predictive (greater odds) of antenatal visit frequency, exclusive breastfeeding knowledge and practices, health-seeking behaviors, and involvement in motherhood support groups; and protective (lower odds) of infant illnesses, breastfeeding challenges, and post-partum depressive symptoms.

**Results:**

Mobile phones were highly prevalent in this rural setting, with 45% (*n* = 79) of post-partum women indicating they owned at least one cell phone. Cell phone owners tended to have higher levels of education (*p* < 0.012) and wealth (*p* < 0.001). Interestingly, mobile phone ownership was only associated with exclusive breastfeeding practices; and phone owners had 75% lower odds of exclusively breastfeeding (adj. OR 0.25; 95% CI: 0.07–0.92, *p* = 0.038) in multivariable models. Though not statistically significant but clinically meaningful, cell phone ownership was associated with fewer depressive symptoms (adj. OR 0.84; 95% CI: 0.39–1.84, p = 0.67) and more social support (adj. OR 1.14; 95% CI: 0.61–2.13, p = 0.70).

**Conclusions:**

Digital literacy and internet connectivity are social determinants of health, thus delving deeper into mothers’ digital experiences to identify and ameliorate their unique barriers to full digital access will be crucial to successful implementation of digital interventions to address post-partum challenges for women in hard-to-reach settings such as ours. Such interventions are of even greater relevance as the Covid-19 pandemic has increased the urgency of reaching vulnerable, marginalized populations.

## Background

In 2014, the World Health Organization (WHO) launched its second Global Health Initiative, calling for affordable and practical technology for low-to-middle-income countries (LMICs) [1, 2]. Since this declaration, the uptake and usage of mobile phone and internet technology across the African continent has burgeoned. The continent has recorded the fastest growth of internet users in the last few years, with users across the region increasing by more than 20 percent year-on-year [3, 4]. Cell phone technology is at the forefront of innovation, and the importance of technology in healthcare is becoming increasingly evident [1, 2, 5]. *Access to mass media and emerging technologies* (e.g., cell phones, the internet, and social media) is a social determinant of health [6] that has been shown to profoundly influence women’s health outcomes [7–9]. In the African region, where women in rural settings with limited access to care, are most vulnerable to maternal mortality and other pregnancy-related morbidities [10, 11], mobile phone access can be an important and life-saving health determinant.

The post-partum period is a time of immense vulnerability in every woman’s reproductive trajectory. Women report drastic physical, social, and emotional changes that if not carefully evaluated and monitored, can have profound detrimental effects on their own and their infants’ health [12, 13]. Though some literature exists on the integration of mobile phone technology and health, few research groups have examined the association between cellular phone ownership and “positive” or expected health behaviors and outcomes; particularly in post-partum mothers in rural African settings, who may be most vulnerable to adverse post-partum outcomes [10]. In this manuscript, we describe the characteristics of post-partum mothers who are mobile phone users in the rural Ntcheu District in the central region of Malawi, and explore how cell phone owners differ from their peers in terms of maternal and infant health practices and outcomes. We hypothesized that cell phone ownership would be associated with greater frequency of antenatal visits, higher exclusive breastfeeding rates and knowledge, more health-seeking behaviors, and greater involvement in motherhood support groups. We also expected and hypothesize that mothers who own cell phones will have fewer reports of breastfeeding problems, infant illnesses, and depressive symptoms.

Understanding the penetration of cell phones and the characteristics, health outcomes, and behaviors of cell phone users in this unique population offers clinicians, researchers, and implementation scientists opportunities to develop contextually appropriate and targeted interventions for a traditionally hard-to-reach yet vulnerable population of women.

## Methods

### Study design

We used a cross-sectional design for this study; where maternal attributes/behaviors and cell phone ownership were assessed at the same time.

### Study setting and data collection

The study was conducted in the Ntcheu District in the central region of Malawi. Participants were recruited from 17 rural villages served by the Gowa Health Center. Recruitment was conducted at the weekly Under 5 Clinics in the Gowa Health Center, as well as the monthly outreach clinics in four outlying villages within the Gowa catchment area, to ensure a representative sample. [14] Mothers, defined as “a female who had given birth to a living infant child, who were 16 years or older and had lived in the same village for at least the past 6 months” were eligible to participate. A brief overview of the research project was given at each clinic, and a convenience sample of mothers of 4 and 5 month old infants who were frequenting the clinics during the study period for their well-child visits, were approached and recruited to participate in the study. After providing written consent, participants were interviewed in their homes at a scheduled, convenient time. Interviews were conducted exclusively with the study participants, without influence from other members of the household. All interviews were conducted by the second (RL) or third (TM) author, along with an experienced Chichewa-speaking Malawian interpreter, who had received study-specific training. In country Institutional Review Board approval was granted by the Malawi National Health Sciences Research Committee, and local village approval was granted by the Ntcheu District Health Officer. The study was also approved by the Institutional Review Board at the University of Rochester.

### Survey questionnaire and measures

The final survey questionnaire was validated for content by local experts and pilot tested prior to deployment. Cell phone ownership was dichotomized based on participants’ response to the question “how many cell phones do you own?” A response of “one” or more were categorized as “owns cell phone” and zero was grouped as “does not own a cell phone”. Because this was a purely quantitative endeavor, we did not collect any qualitative data on the participants’ online behaviors or user experiences. Participants were asked their age, marital status, number of living children, educational attainment, household size, visits to the antenatal clinic, breastfeeding practices in the last four weeks, breastfeeding knowledge, troubles with breastfeeding, and care-seeking behaviors for their breastfeeding problems. Participants were also inquired about recent common illnesses with their infants (cough, malaria, fever and diarrhea) and their health seeking behaviors for these illnesses. Their involvement with motherhood support groups was also asked. Child’s age was calculated based on the medical charts and/or providers’ information.

Food insecurity, depression and wealth were assessed using validated questionnaires, specific for this population. Assessments of food insecurity, which is a salient issue in rural Malawi [14–16], was measured using a modified, Malawi-specific Household Food Insecurity Access Scale (HFIAS) from the USAID [17, 18]. Total scores on the measure range from 0 to 40. High food insecurity was defined as a score of 9 or higher on the scale [18]. Post-partum depression symptomatology was measured by using a validated Chichewa version of the Self-Reporting Questionnaire (SRQ) depression screener [19]. This measure was specifically designed to evaluate clinical depression in the rural Malawian population. It has been validated to detect clinical depression in this vulnerable population and deemed culturally and contextually appropriate. The SRQ is comprised of 20 questions regarding the participants’ feelings, thoughts, and emotions in the past four weeks. Responses are coded as ‘yes’ or ‘no’ for each question and scored on a scale of 0 to 20. As has been done in previous literature, high-risk for clinical depression was defined as having a score of 8 or higher on the SRQ [19]. Household wealth was assessed using the weighted asset-based approach proposed by Morris et al. for use in rural African populations [20]. This approach assumes that households with greater resources will purchase and own a greater number of consumer durables. The scale was dichotomized to represent low (≤ 0.32 score) or high wealth (> 0.32) in our analyses. Given the study’s small sample sizes, and because education and wealth were highly correlated (R = 0.54; *p* < 0.0001), we adjusted for education only in multivariable models, as has been done in similarly published studies [9].

### Statistical analyses

Comparisons were made between post-partum mothers who own cell phones and those who do not, using descriptive statistics, t-tests and χ2 tests as appropriate. Logistic regression estimates were used to determine the odds of each outcome, predicted by cell phone ownership. As previously stated, we adjusted for education only in multivariable models. Statistical significance for all final effect estimates and interpretations was determined using the traditional *p* < 0.05 cut-off. Data was analysed using the statistical software SAS® software (SAS Institute Inc., Cary, NC, USA).

## Results

All women who were approached for participation agreed and consented to be a part of the study. We recruited and consented 174 women to participate in the study (Table 1). The women ranged from 16 to 60 years of age, and had a mean age of 25.4 (± 7.5 SD). The average age of their infants was 147 days (± 14.3 SD). Cell phone penetration was highly prevalent in the study sample, with 45% (*n* = 79) of post-partum women indicating they owned at least one cell phone. Cell phone ownership was more common amongst those in their 20’s (40.5%) and 30’s (27.8%) than in the adolescent age group (25.3%). There were statistically significant differences in the educational status and wealth attainment of cell phone users. Women who owned mobile phones tended to have at least a high school education (*p* = 0.006) and tended to be wealthier (*p* < 0.001). There were no statistically significant differences between mobile phone owners and non-owners in terms of marital status, living children or household size. Food insecurity scores were lower amongst those with cell phones than those without, though this finding was marginally significant (*p* = 0.09).

**Table 1:** Sociodemographic characteristics of study participants, stratified by cell phone ownership (*n* = 174)

Demographic Characteristic	Owns Cell Phone (*n* = *79*)	Does not Own a Cell Phone (*n* = *95*)	Total Participants *(n* = *174)*
***Maternal Age group (p* = *0.57)*** **Range 16-60yrs** **Mean 25.4 (± 7.5 SD)**			
Adolescent (16–19)	20 (25.3%)	19 (20%)	39 (22.4%)
Young Twenties (20–29)	32 (40.5%)	43(45.3%)	75 (43.1%)
Older (≥ 30)	22 (27.8%)	32 (33.7%)	54 (31.0%)
Missing	5 (6.3%)	1 (1.1%)	7 (4.0%)
***Married (p* = *0.98)***			
Yes	65 (82.3%)	78 (82.1%)	143 (82.0%)
No	14 (17.7%)	17 (17.9%)	31 (18.0%)
***Current Child Age group (p* = *0.56)*** **Range 123–180 days** **Mean 147.1 (± 14.3 SD)**			
≤ 151 days (≤ 4 months)	50 (63.3%)	56 (58.95%)	106 (60.9%)
> 151 days (> 4 months)	29 (36.7%)	39 (41.05%)	68 (39.1%)
***Number of living children (p* = *0.96)*** **Range 1–8** **Mean 2.7 (± 1.8 SD)**			
< 3 children	46 (58.2%)	55 (57.9%)	101(58.0%)
≥ 3 children	33 (41.8%)	40 (42.1%)	73 (42.0%)
*Maternal Education Level (p* = 0.006)*			
High Level of Education (US middle school or greater (standard 6 to form 4))	59 (74.7%)	52 (54.7%)	111 (63.8%)
Lower Level of Education (US elementary to no formal education (Standard 1 to 5 or no formal education))	20 (25.3%)	43 (45.3%)	63 (36.2%)
*Household wealth (p* < 0.001)* **Range 0–6.7** **Mean 0.4 (± 0.71 SD)**			
High Household wealth^a^	45 (57.0%)	16 (16.8%)	61 (35.0%)
Low Household wealth^a^	34 (43.0%)	79 (83.2%)	113 (65.0%)
***Household size (p* = *0.18)*** **Range 1–15** **Mean 5.0 (± 2.0 SD)**			
≤ 4 in household	31 (39.2%)	47 (49.5%)	78 (44.8%)
> 4 in household	48 (60.8%)	48 (50.5%)	96 (55.2%)
***Food Insecurity Score (p* = *0.09)*** **Range 0–31** **Mean 10.4 (± 8.1 SD)**			
High (Score ≥ 9 on HFIAS)	34 (43.0%)	53 (55.8%)	87 (50.0%)
Low (Score < 9 on HFIAS)	45 (57.0%)	42 (44.2%)	87 (50.0%)

^a^High household wealth defined as a score greater than 0.32, and low household wealth defined as 0.32 or lower on the scale [20]

^*^Statistically significant at *p* < 0.05 level

In terms of health behavior and outcomes (Table 2), cell phone ownership was only statistically associated with decreased odds of exclusive breastfeeding in multivariable models. Women who owned mobile phones had 75% reduced odds of exclusive breastfeeding (EBF) (adj. OR 0.25; 95% CI: 0.07–0.92, *p* = 0.038), even though the direction of the effect estimate suggests that they were more knowledgeable of the WHO’s recommendation of 6 months of EBF (adj. OR 1.35, 95% CI:0.57–3.21; *p* = 0.50); though this finding did not reach statistical significance. However cell phone owning mothers tended to report more breastfeeding challenges (adj. OR 1.31, 95% CI: 0.70–2.50; *p* = 0.40).

**Table 2:** Health Behaviors and Outcomes associated with Cell Phone Ownership (*n* = 174). Effect estimate is Odds of Outcome predicted by Cell Phone Ownership (Yes vs No(ref))

**Behavior/Outcome**	**Owns Cell Phone** **(*n* = *79*)**	**Does not Own a Cell Phone** **(*n* = *95*)**		**Total** ***(n* = *174)***	**Crude** **Odds Ratio, 95% CI**	**Education-adjusted** **Odds Ratio, 95% CI**
***Number of Antenatal Visits for current child’s pregnancy*** **Range 1–8** **Mean 3.9 (± 1.09 SD)**						
≥ 4 visits	50 (63.3%)	67 (70.5%)	*(p* = *0.31)*	117 (67.2%)	0.72 (0.38–1.36; *p* = 0.31)	0.69 (0.36–1.32; *p* = 0.26)
≤ 3 visits (ref)	29 (36.7%)	28 (29.5%)		57 (32.8%)		
***Exclusively Breastfeeding current child***						
Yes	3 (4.5%)	12 (15.2%)		15 (10.3%)	0.26 (0.07–0.97; *p* = 0.045)*	0.25 (0.07–0.92; *p* = 0.038)*
No (ref)	64 (95.5%)	67 (84.8%)	*(p* = 0.03)*	131 (89.7%)		
Missing**	12 (15.2%)	16 (16.8%)		28 (16.1%)		
***Knows to exclusively breastfeeding for at least 6 months)***						
Yes	69 (87.3%)	78 (82.1%)		147 (84.5%)	1.50 (0.65–3.50; *p* = 0.34)	1.35 (0.57–3.21; *p* = 0.50)
No (ref)	10 (12.7%)	17 (17.9%)	*(p* = *0.34)*	27 (15.5%)		
***Problems with Breastfeeding in last 4 weeks)***						
Yes	33 (41.8%)	33 (34.7%)	*(p* = *0.34)*	66 (37.9%)	1.35 (0.73–2.50; *p* = 0.34)	1.31 (0.70–2.50; *p* = 0.40)
No (ref)	46 (58.2%)	62 (65.3%)		108 (62.1%)		
***Discussed Breastfeeding problems with healthcare provider (amongst those with problems only, n = 66)***						
	*n* = 33	*n* = 33		66		
Yes	11 (35.5%)	12 (37.5%)		23 (36.5%)	0.92 (0.33–2.56; *p* = 0.87)	0.75 (0.26–2.21; *p* = 0.60)
No (ref)	20 (64.5%)	20 (62.5%)	*(p* = *0.87)*	40 (63.5%)		
Missing**	2 (6.5%)	1 (3.1%)		3 (4.5%)		
***In last 4 weeks, child has had any diarrhea, cough with trouble breathing, malaria or fever***						
Yes	63 (79.8%)	70 (73.7%)	*(p* = *0.35)*	133 (76.4%)	1.41 (0.69–2.87; *p* = 0.35)	1.38 (0.67–2.86; *p* = 0.39)
No (ref)	16 (20.3%)	25 (26.3%)		41 (23.6%)		
***Sought treatment for child’s illness (amongst those with problems only, n = 133)***						
	*n* = 63	*n* = 70		133		
Yes	50 (79.4%)	51 (72.9%)	*(p* = *0.38)*	101(75.9%)	1.43 (0.64–3.21; *p* = 0.38)	1.31 (0.57–3.00; *p* = 0.52)
No (ref)	13 (20.6%)	19 (27.1%)		32 (24.1%)		
***Depressive Symptoms in post-partum period***						
Yes, meets clinical depression cutoff (score ≥ 8 on SRQ)	14 (17.7%)	20 (21.1%)	*(p* = *0.58)*	34 (19.5%)	0.81 (0.38–1.73; *p* = 0.58)	0.84 (0.39–1.84; *p* = 0.67)
No, does not meet clinical depression cutoff on SRQ (score < 8) (ref)	65 (82.3%)	75 (79.0%)		140 (80.5%)		
***Involved in any Motherhood Support Groups***						
Yes	47 (60.3%)	56 (59.0%)		103 (59.5%)	1.06 (0.57–1.94; *p* = 0.86)	1.14 (0.61–2.13; *p* = 0.70)
No (ref)	31 (39.7%)	39 (41.1%)	*(p* = *0.86)*	70 (40.5%)		
Missing**	1(1.3%)	0 (0.0%)		1 (0.6%)		

^*^Statistically significant at *p* < 0.05 level

^**^Missing not included in effect estimates

Other outcomes and behaviors of interest that were not statistically significant but were in the expected hypothesized direction included positive health seeking behaviors for child (adj. OR: 1.31, 95% CI: 0.57–3.00; *p* = 0.52), reduced odds of post-partum depression (adj. OR: 0.84; 95% CI: 0.39–1.84, *p* = 0.67) and greater involvement in motherhood support groups (adj. OR 1.14; 95% CI: 0.61–2.13, *p* = 0.70).

## Discussion

To our knowledge, this is the first study to evaluate cell phone access and associated health behaviors and outcomes amongst post-partum mothers in Malawi. The 45% prevalence rate of cell phone ownership in this post-partum population of rural Malawian mothers was higher than the 38% cell phone penetration rate reported for the African region [3, 21, 22]. Cell phone ownership was associated with greater educational attainment and wealth, paralleling recent reports by the Pew Research Center [22]. In a study of 6 African countries (Ghana, Kenya, Nigeria, Senegal, Tanzania and South Africa), survey responders with at least a secondary/high school education were more likely to own mobile phones, compared to those with less education. Our study findings are in line with these reports, which suggest that even in rural and remote settings, *access* to important social determinants of health such as mass media and emerging technologies, is influenced by within-population differences in education and wealth. Public health programs that use cell phone technology for interventions in Africa should, at the onset, address the unequal access to mobile phones inherent within these populations. This issue has gained greater saliency in recent times, as countries ramp up efforts to vaccinate large masses of their populations against the coronavirus (Covid-19) [23]. Public health systems may need to rely on mobile phones and other forms of Information and communication technologies (ICTs) to reach the most vulnerable, hard-to-reach sectors of populations. Knowing the mobile phone access and penetration rates will be key to curbing vaccine misinformation, reaching vaccination targets and addressing inequities in vaccine access. To this end, it is crucial to understand what owning a cell phone means for mothers in this setting. Simply owning a cell phone does not mean one has sufficient *digital literacy* or internet/data access to the device. Are these mothers able to use their cell phones to obtain the health information they need for themselves and their infants (e.g. where and how to access the Covid-19 vaccine? Information about vaccine safety for pregnant women and infants? etc.)? Do they have adequate internet or data connection to *access* that information or is digital access a barrier to effective usage? Digital literacy and internet connectivity are social determinants of health, [6] thus delving deeper into mothers’ digital experiences to identify and ameliorate their unique barriers to full digital access will be crucial to successful implementation of digital interventions in such vulnerable populations. In rural settings such as that of our study participants’, cell phone owners may have to strike a balance between their mobile phone usage and the lack of continuous electrical power to keep devices fully operational. Heavy cell phone use is often associated with rapid depletion of battery power, thus access to solar and/or constant sources of power in such settings, should be a critical consideration for public health programs that incorporate ICTs. Mobile data is another concern that implementation researchers should bear in mind; particularly in the nascent African market. Despite the rapidly expanding mobile phone market, getting online is expensive for many. Purchasing a handset and 500 MB of data costs an average 10 percent of monthly income; double the five percent threshold recommended by the United Nations Broadband Commission [4]. Cell phone owners may be hindered from accessing necessary online content by wealth-driven limitations such as mobile data. Because we sought to quantitatively evaluate whether cell phone ownership (yes/no) was associated with post-partum health outcomes and behaviors, we did not qualitatively assess participants' online behaviors or user experiences (e.g. internet connectivity, mobile data availability, digital literacy (websites visited, health information sought), etc.). A study that evaluates whether and how new mothers use their phones to seek health information, their levels of digital literacy as well their digital access, would further enhance the findings of the current study.

With regards to maternal behaviors and outcomes, cell phone owners in our study surprisingly had lower rates of EBF than their peers. These women were also more likely to report-albeit statistically insignificant-that they had recently experienced difficulties with breastfeeding, offering a potential explanation for the group’s low breastfeeding rates (i.e. women who experience breastfeeding difficulties are less likely to breastfeed). These findings however, are in contrast to the literature on mobile phone use and breastfeeding rates. In a two armed, prospective study of pregnant women through the post-partum phase, Patel and team [24] showed that women who used mobile phones were 6 times more likely to exclusively breastfeed than than those in the control arm (AOR [95% CI]: 6.3 [4.9–8.0]). Similar results were reported by Jerin et al., [25] in a population of Bangladeshi women. Additional clinical trials of mothers in Nigeria, China, and Kenya [9] also support the finding that SMS/cell phone interventions improve EBF rates up to 4 months post-partum. In their meta analyses, Lee and co [9] show consistently that i) EBF initiation on hour after birth (OR 2.01, 95% CI 1.27–2.75, I^2^ = 80.9%); ii)3-4 months post-partum (OR 1.88, 95% CI 1.26–2.50, I^2^ = 52.8%) and iii) 6 six months post-partum (OR 2.58, 95% CI 1.44–3.71, I^2^ = 0.0%) are higher in SMS/cell phone intervention groups than their peers. Every year, nearly one million deaths occur due to suboptimal breastfeeding. If universally practiced, exclusive breastfeeding alone prevents 11.6% of all under 5 deaths. [24] In our study, since infants born to moms in the cell phone group were less likely to receive breastmilk at full capacity, they were also more prone to respiratory issues, diarrhea, malaria, and other endemic childhood illnesses (again statistically insignificant but clinically important direction of effect estimate). Breastmilk has significant immune benefits [8, 9, 12] that protect infants against common childhood illnesses. Reports also show that women who breastfeed have better mental health outcomes compared to those who do not breastfeed [8, 12, 26]. Interventions that use cell phones to address breastfeeding challenges of post-partum mothers have the potential to be mutually beneficial for both mothers and their infants. Our results suggest that there are opportunities for implementation scientists to use cell phone technology to bridge the patient/provider gap for post-partum mothers and obstetric providers (physicians, nurses, midwives, doulas and lactation specialists), to address breastfeeding challenges, and improve maternal/child health outcomes in the vulnerable post-partum phase of women’s reproductive-and parenting-spectrum. In our study population, mothers with cell phones were more knowledgeable of EBF practices. Mobile phone applications (apps) or digital programs that help mothers translate that knowledge into practice by connecting them to lactation specialists for example, could positively impact breastfeeding outcomes.

Although not statistically significant but clinically relevant and worth noting, is the association between cell phone ownership, reduced post-partum depression, and greater involvement in motherhood support groups. These findings align with research from other parts of Africa that show a positive association between cell phone use and other aspects of women’s/maternal health such as contraceptive use (Burkina Faso), antenatal care attendance (Tanzania), skilled attendance at delivery (Tanzania), and overall maternal healthcare utilization (Nigeria) (to name a few) [7, 9]. The association between mobile phone use and reduced post-partum depression is especially salient because in a resource-limited setting like Malawi, where food insecurity is highly prevalent, depressive symptoms in pregnant and post-partum women cannot be ignored, as their impact on maternal/infant morbidity and mortality outcomes can be devastating [14, 27–29]. Cell phones provide users access to online support systems, mood modifying materials and other pertinent online content such as entertainment, social media interactions, texting, and other mood modifying content [3, 21, 22]. They can be used to reduce social isolation and enhance one’s sense of social connectedness. Postpartum depression is complex not only because of the surge of reproductive hormones, but also because of social pressures and gendered expectations of women, especially those in rural settings such as Malawi [30]. Access to cell phones can connect women in such settings with virtual social networks that may be beyond the realm of their physical settings [31] in coping with external stressors. This is especially important for women of Caribbean and African origin [32], who may have limited access to psychological interventions after birth. Mobile phones offer researchers ample opportunity to create and/or tailor culturally appropriate virtual interventions to serve the unique needs of this subpopulation of women and mothers, who may experience mental health inequities [33].

Even though this study is, to our knowledge, the first to evaluate cell phone ownership and post-partum health outcomes and behaviors in Malawian women, it is not without limitations. The primary limitation of our study is in its’ design. As a cross-sectional design, the study lacks temporality and limits our ability to hypothesize on the associations between cell phone ownership and time-varying factors such as maternal age and education. The adolescents in our study for example, had yet to reach their full educational (and wealth) potential; thus, they had lower cell phone ownership than those in their 20’s and 30’s who were more educated and had greater access to financial capital. It will be interesting to follow this adolescent cohort prospectively, to determine if those who are able to continue their education i) also acquire more digital literacy ii) gain more digital access and iii) if and how greater digital literacy and access modify their health behaviors and outcomes; as social determinants of health. The study design also inhibits our ability to make any causal inferences regarding the relationship between cell phone ownership and mood and social interaction. Though we are assuming that cell phone ownership predicts these two outcomes, it is possible that less depressed mothers and/or those who are more socially adept are more inclined to engage in mood-enhancing behaviors and thus seek cell phone ownership. Our sampling scheme further limits our ability to understand the relationships between these variable as it excluded mothers who were unable to attend the Gowa Health Clinic. Though we made all attempts to capture a representative sample within the Gowa catchment area who the study can be generalized to, mothers who were unable to participate in the study may represent a more vulnerable and potentially depressed subgroup within the community, who may be under-represented in our study population. Even though we had a sample size of 174 new mothers partaking in the study, more vulnerable women may not have been able to take part. Thus a more inclusive study may be warranted. A larger study may also be needed because although a number of odds ratios were in expected directions, they were not statistically significant. A larger, well-powered study is needed to detect statistically meaningful differences in the association between mobile phone and maternal post-partum behaviors. Without a prospectively designed study to follow mobile phone user and non-users in larger and more representative populations over time, researchers are unable to make causal assumptions about the effects of cell phone ownership on mood and social engagement. The study should not be generalized to populations beyond the Gowa catchment area. Future studies should *prospectively* track how cell phone ownership influences health outcomes and behaviors for heterogeneous populations over time, as well as evaluate the association between cell phone ownership, digital literacy, and digital access.

## Conclusions

In the African region, where non-negligible subsets of populations reside in rural settings, mobile phone access offers unique opportunities for public health scientists to communicate and engage with hard-to-reach and often vulnerable populations, with unique health challenges. Understanding what “cell phone ownership” means to mothers, and utilizing mobile phone technology and other ICTs to help mothers translate their knowledge into practice could have positive impacts on maternal and infant health outcomes. Such efforts are critical in this post-pandemic digital era.

## Data Availability

The datasets generated and/or analyzed during the current study are not publicly available because they are proprietary but are available from the corresponding author on reasonable request.
